# Development and Characterization of a cDNA-Launch Recombinant Simian Hemorrhagic Fever Virus Expressing Enhanced Green Fluorescent Protein: ORF 2b’ Is Not Required for In Vitro Virus Replication

**DOI:** 10.3390/v13040632

**Published:** 2021-04-07

**Authors:** Yingyun Cai, Shuiqing Yu, Ying Fang, Laura Bollinger, Yanhua Li, Michael Lauck, Elena N. Postnikova, Steven Mazur, Reed F. Johnson, Courtney L. Finch, Sheli R. Radoshitzky, Gustavo Palacios, Thomas C. Friedrich, Tony L. Goldberg, David H. O’Connor, Peter B. Jahrling, Jens H. Kuhn

**Affiliations:** 1 Integrated Research Facility at Fort Detrick, National Institute of Allergy and Infectious Diseases, National Institutes of Health, Fort Detrick, Frederick, MD 21702, USA; caiyingyun@yahoo.com (Y.C.); shuiqing.yu@nih.gov (S.Y.); bolobs1@hotmail.com (L.B.); elena.postnikova2@nih.gov (E.N.P.); steven.mazur@nih.gov (S.M.); johnsonreed@niaid.nih.gov (R.F.J.); courtney.finch@nih.gov (C.L.F.); jahrlingp@niaid.nih.gov (P.B.J.); 2 College of Veterinary Medicine, Kansas State University, Manhattan, KS 66506, USA; yfang@vet.k-state.edu (Y.F.); lyhliyanhua@gmail.com (Y.L.); 3 Department of Pathology and Laboratory Medicine, School of Medicine and Public Health, University of Wisconsin–Madison, Madison, WI 53705, USA; michaellauck@gmail.com (M.L.); thomasf@primate.wisc.edu (T.C.F.); dhoconno@wisc.edu (D.H.O.); 4 Emerging Infectious Pathogens Section, National Institute of Allergy and Infectious Diseases, National Institutes of Health, Fort Detrick, Frederick, MD 21702, USA; 5 United States Army Medical Research Institute of Infectious Diseases, Fort Detrick, Frederick, MD 21702, USA; sheli.r.radoshitzky.ctr@mail.mil (S.R.R.); gustavo.f.palacios.civ@mail.mil (G.P.); 6 Department of Pathobiological Sciences, School of Veterinary Medicine, University of Wisconsin–Madison, Madison, WI 53706, USA; tony.goldberg@wisc.edu; 7 Wisconsin National Primate Research Center, University of Wisconsin–Madison, Madison, WI 53715, USA

**Keywords:** *Arteriviridae*, cell tropism, infectious clone, *Nidovirales*, reverse genetics, SHFV, simarterivirin, simarterivirus, *Simarterivirinae*, simian hemorrhagic fever virus

## Abstract

Simian hemorrhagic fever virus (SHFV) causes acute, lethal disease in macaques. We developed a single-plasmid cDNA-launch infectious clone of SHFV (rSHFV) and modified the clone to rescue an enhanced green fluorescent protein-expressing rSHFV-eGFP that can be used for rapid and quantitative detection of infection. SHFV has a narrow cell tropism in vitro, with only the grivet MA-104 cell line and a few other grivet cell lines being susceptible to virion entry and permissive to infection. Using rSHFV-eGFP, we demonstrate that one cricetid rodent cell line and three ape cell lines also fully support SHFV replication, whereas 55 human cell lines, 11 bat cell lines, and three rodent cells do not. Interestingly, some human and other mammalian cell lines apparently resistant to SHFV infection are permissive after transfection with the rSHFV-eGFP cDNA-launch plasmid. To further demonstrate the investigative potential of the infectious clone system, we introduced stop codons into eight viral open reading frames (ORFs). This approach suggested that at least one ORF, ORF 2b’, is dispensable for SHFV in vitro replication. Our proof-of-principle experiments indicated that rSHFV-eGFP is a useful tool for illuminating the understudied molecular biology of SHFV.

## 1. Introduction

Simian hemorrhagic fever (SHF) was first identified in 1964 as an acute, almost uniformly lethal disease of Asian macaques (*Macaca* spp.) in captive colonies in Sukhumi, Georgian Soviet Socialist Republic, USSR, and Bethesda, Maryland, USA [1,2,3]. Three distinct viruses—today classified in the subfamily *Simarterivirinae* (*Nidovirales*: *Arteriviridae*)—have been identified as causative agents of SHF: simian hemorrhagic encephalitis virus (SHEV), the etiological cause of the Sukhumi epizootic [4]; simian hemorrhagic fever virus (SHFV), the causative agent of the Bethesda epizootic [5]; and Pebjah virus (PBJV), which caused an outbreak in 1989 in a primate-holding facility in Alamogordo, New Mexico, USA [4,6,7]. The natural reservoirs of all three viruses remain to be identified but are likely to be African primates.

Evidence indicates that simarterivirins (“-virins” = members of a subfamily “-*virinae*” [8]) are widely distributed among African primates. For example, numerous SHFV epizootics occurred in primate facilities during the period 1964–1996 (reviewed in [4]). Kibale red colobus virus 1 (KRCV-1) was discovered in apparently healthy red colobus (*Procolobus* [*Piliocolobus*] *rufomitratus tephrosceles* Elliot 1907) sampled in 2010 in Kibale National Park, Uganda [9]; it is a simarterivirin that causes mild SHF-like disease in experimentally inoculated crab-eating macaques (*Macaca fascicularis* Raffles, 1821) [10]. In addition to SHEV, SHFV, PBJV, and KRCV-1, at least seven more simarterivirins have been discovered, often at high titers and sometimes in the presence of other simarterivirins, in serum of apparently healthy African primates of numerous species [9,11,12,13,14]. Many of these simarterivirins may cause SHFV or similar diseases once introduced into a non-natural host such as macaques or, potentially, humans [15].

SHFV is the only fatal SHF-causing simarterivirin that has been isolated in cell culture [5,16]. Consequently, SHF pathogenesis due to SHFV infection has been characterized in greatest detail. SHF caused by SHFV presents with sudden onset of fever, anorexia, edema, dyspnea, diarrhea, lymphadenopathy, splenomegaly, hemorrhage, disseminated intravascular coagulation, liver and adrenal gland necrosis, lymphocyte depletion, and systemic shock [3,10,17,18,19,20]. SHFV initially targets macrophages but also has been detected in astrocytes, vascular endothelial cells, glial cells, and neuronal cell bodies [21]. However, growth of SHFV in vitro has been successful only in primary macaque and baboon macrophages and myeloid dendritic cells and in a few grivet (*Chlorocebus aethiops* (Linnaeus, 1758)) kidney cell lines (i.e., MA-104, BS-C-1, MARC-145, and CL 2621, but not Vero or Vero E6) [5,18,21,22]. These findings suggest that only some primate cell lines are susceptible to SHFV particle entry and/or, once inside the cell, permissive to SHFV replication and particle formation/egress.

Simarterivirions are spherical enveloped particles that contain linear, unsegmented, positive-sense RNA genomes that are highly similar in organization. The 5′ half of the simarterivirin genome serves directly as a ribosomal substrate. This part of the genome contains two large open reading frames (ORF 1a and ORF 1b) in separate reading frames that encode polyproteins pp1a and pp1ab (expressed via ribosomal frameshifting by merging of ORF 1a and ORF 1b). During or following biosynthesis, these polyproteins are auto-cleaved into at least 12 non-structural proteins required for virus replication and transcription [23,24,25]. The 3′ half of the genome contains several main ORFs (ORFs 2–7) that are transcribed into a coterminal nested set of subgenomic RNAs serving as templates for the transcription of an equally nested set of mRNAs that generate structural proteins [23,26,27,28]. Two major structural envelope proteins, the non-glycosylated matrix protein (M, encoded by ORF 6) and glycoprotein 5 (GP5, encoded by ORF 5) form heterodimers that function in an unknown way in virion assembly and possibly cell entry, and which also contain major immune epitopes [26,27,29,30]. The third major structural protein, the nucleocapsid protein (N, encoded by ORF 7) encapsidates the viral genome [28,31]. Three minor structural proteins (GP2, GP3, and GP4 encoded by ORF 2b, ORF 3, and ORF 4, respectively) form a heterotrimeric complex [28,32]. This complex is inserted into the virion envelope by the minor structural envelope protein (E, encoded by ORF 2a) that also may function as an ion channel during virion entry [28,32,33]. Unlike arterivirids (“-virids” = members of a family “-*viridae*” [8]) of other subfamilies, simarterivirins encode and express a second set of minor structural proteins: GP2′ (encoded by ORF 2a’), GP3′ (encoded by ORF 3′), GP4′ (encoded by ORF 4′), and E’ (encoded by ORF 2b’) [27]. The GP2-GP3-GP4 complex is hypothesized to mediate virion entry, which, at least in the case of SHFV, occurs after interaction with a proteinaceous receptor, which may be CD163, via actin-independent, but dynamin-dependent and clathrin-mediated, endocytosis [28]. The functions of GP2′, GP3′, GP4′, and E’ are unknown.

Because SHEV, SHFV, PBJV, and KRCV-1 are neglected animal pathogens, few tools and detection reagents are available for their characterization. In this study, we developed a recombinant cDNA-launch infectious clone of SHFV that expresses a reporter protein (enhanced green fluorescent protein [eGFP]). In proof-of-principle experiments, we used this rSHFV-eGFP to further characterize SHFV cellular tropism and to ascertain whether any SHFV minor structural proteins are dispensable for in vitro replication.

## 2. Materials and Methods

### 2.1. Cell Lines

Grivet (*Chlorocebus aethiops* (Linnaeus, 1758)) kidney epithelial BS-C-1 cells (American Type Culture Collection (ATCC), Manassas, VA, USA; #CCL-26), embryonic grivet kidney MA-104 cells (ATCC, #CCL-2378), and MARC-145 cells (provided by Kay Faaberg, U.S. Department of Agriculture, National Animal Disease Center, Ames, IA, USA), primary western gorilla (*Gorilla gorilla* (Savage, 1847)) RPGor53 and common chimpanzee (*Pan troglodytes* (Blumenbach, 1775)) S008397 and RP00226 fibroblasts (Coriell Institute for Medical Research, Camden, NJ, USA), and hispid cotton rat (*Sigmodon hispidus* Say and Ord, 1825) lung CRL cells (ATCC; #PTA-3920) were maintained in Eagle’s minimum essential medium (EMEM; Thermo Fisher Scientific, Waltham, MA, USA) supplemented with 10% heat-inactivated fetal bovine serum (FBS; Sigma-Aldrich, St. Louis, MO, USA).

Human embryonic kidney epithelial HEK293T/T17 cells (ATCC; #CRL-11268), human cervical epithelial HeLa cells (ATCC; #CCL-2), grivet kidney epithelial Vero cells (ATCC; #CCL-81), Vero E6 cells (BEI Resources, Manassas, VA, USA; #NR696,), baby golden hamster (*Mesocricetus auratus* Waterhouse, 1839) kidney BHK-21 fibroblasts (ATCC; #CCL-10), striped dwarf hamster (*Cricetulus barabensis* (Pallas, 1773)) ovary CHO-K1 cells (ATCC; #CCL-61), house mouse (*Mus musculus* Linnaeus, 1758) embryonic NIH 3T3 fibroblasts (ATCC; #CRL-1658), Brazilian free-tailed bat (*Tadarida brasiliensis* (I. Geoffroy, 1824)) adult lung Tb1 Lu cells (ATCC; #CCL-88), eastern pipistrelle (*Pipistrellus subflavus* (F. Cuvier, 1832)) adult lung PESU-B5L cells [34] (provided by Eric F. Donaldson, University of North Carolina at Chapel Hill, Chapel Hill, NC, USA), African straw-colored fruit bat (*Eidolon helvum* Kerr, 1792) adult kidney EidNi/41.3 cells [35], Büttikofer’s epauletted fruit bat (*Epomops buettikoferi* (Matschie, 1899)) adult kidney EpoNi/22.1 cells, Daubenton’s myotis (*Myotis daubentonii* (Kuhl, 1817)) adult lung MyDauLu/47.1 cells, Egyptian rousette (*Rousettus aegyptiacus* (E. Geoffroy, 1810)) adult kidney RoNi7.1 cells, hammer-headed fruit bat (*Hypsignathus monstrosus* H. Allen, 1861) fetal lung HypLu/45.1 cells, and Egyptian rousette adult kidney RoNi/7.2 cells [36] (all generated with funds from the German Research Council (DR 772/10-2) and provided by Marcel A. Müller and Christian Drosten, Charité–Universitätsmedizin Berlin, Berlin, Germany) were grown in Dulbecco’s modified Eagle’s medium (DMEM; Thermo Fisher Scientific) supplemented with 10% heat-inactivated FBS.

Egyptian rousette embryo Ro5T and Ro6E cells [37] (provided by Ingo Jordan, ProBioGen AG, Berlin, Germany) and hammer-headed fruit bat fetal kidney HypNi/1.1 cells [38] (generated with funds from the German Research Council (DR 772/10-2) and provided by Marcel A. Müller and Christian Drosten, Charité–Universitätsmedizin Berlin, Berlin, Germany) were grown in DMEM/F-12 (Lonza, Walkersville, MD, USA) supplemented with 10% heat-inactivated FBS.

The “NCI-60 panel” of highly characterized human cancer cell lines [39] was obtained from the National Cancer Institute’s Developmental Therapeutics Program (NCI DTP; Frederick, MD, USA). For this study, 53 adherent cell lines of this panel were used and grown in Roswell Park Memorial Institute 1640 medium (RPMI-1640; Thermo Fisher Scientific) supplemented with 10% heat-inactivated FBS as previously described [40].

All cells were grown at 37 °C in a humidified 5% CO_2_ atmosphere.

### 2.2. Viruses

SHFV variant NIH LVR42-0/M6941 (ATCC; #VR-533) was propagated in MA-104 cells as previously described [28]. Equine arteritis virus (EAV) strain Bucyrus was obtained from ATCC (#VR-796) and propagated in BHK-21 cells as previously described [41].

### 2.3. Plasmid Construction

At the time of cloning, a single SHFV genome sequence, that of an isolate of variant NIH LVR42-0/M6941, was available in GenBank (GenBank #AF180391.1; RefSeq #NC_003092.1). This genome was commercially synthesized (Geneart, Regensburg, Germany), flanked by a human cytomegalovirus (CMV) immediate early enhancer-containing promoter at the 5′ end and a hepatitis delta virus 1 (HDV-1) ribozyme sequence at the 3′ end, and then assembled into the pACYC177 vector backbone (designated “pCMV-SHFV-NC_003092.2”), which had previously been used to successfully rescue another arterivirid, porcine reproductive and respiratory syndrome virus 1 (PRRSV-1) [42,43]. To ensure correct sequence, we acquired the commercially available SHFV variant NIH LVR42-0/M6941. We propagated this isolate, designated KS_06_17_11, in cell culture and sequenced its genome using high-throughput sequencing (GenBank #KM373784) [16]. The differences between the original GenBank genome sequence and the consensus sequence of isolate KS_06_17_11 are shown in Table 1.

Using standard site-directed mutagenesis and Gibson assembly, we then changed pCMV-SHFV-NC_00309.2 to encode isolate KS_06_17_11 (with the exception of silent mutations at positions 1658, 3785, 4277, and 4895, which are indicated in grey in Table 1). This plasmid was designated “pCMV-SHFV.”

To generate a reporter-encoding SHFV, we inserted a gene cassette encoding eGFP between ORF 1b and ORF 2a’ of rSHFV under the control of a 2/2a transcription regulation sequence (TRS) at nsp12. The stop codon of the eGFP ORF was extended by the TRS of ORF 5 (TRS5) to provide the transcription activation sequence for the expression of ORF 2a’, ORF 2b’, and ORF 3′ (designated “pCMV-SHFV-eGFP”). To achieve this, pCMV-SHFV was digested with restriction endonuclease *Eco*RI (New England Biolabs, Ipswich, MA, USA). Three fragments were amplified by PCR. Fragment 1, which contained SHFV genome nucleotides 8389–10996, was amplified with primers 5′SHFV 8389-F-*Eco*RI (5′-TGGACCTGCCGCTTTCAGGG-3′) and 3′SHFV-10996-R eGFP+*Not*I (5′-TCCTCGCCCTTGCTCACCATTGCGGCCGCATTAAGGAGTACCAACAGTAGGATT-3′), with pCMV-SHFV as a template. Fragment 2, which contained the eGFP ORF, was amplified with primers 5′-eGFP-F (5′-ATGGTGAGCAAGGGCGAGGAG-3′) and eGFP-R TRS5 + *Spe*I (5′ GAGTCGCGCAATCACAATGAACAAGGTTATGATGACTAGTTTACTTGTACAGCTCGTCCA 3′), with plasmid pWIZ-eGFP (Genlantis, San Diego, CA, USA) as a template. Fragment 3, which contained SHFV genome nucleotides 10953–13628, was amplified with primers SHFV 10953-F TRS5 (5′-TCATTGTGATTGCGCGACTCCGCTCCTTAACTACCTAATTATGAGTTTCTGTCCAGGTTT-3′) and SHFV 13628-R *Eco*RI (5′-TCAAGCAAGTTTGATCCCCG-3′), with pCMV-SHFV as a template. These three fragments, together with *Eco*RI-digested pCMV-SHFV, were Gibson-assembled according to the manufacture’s instruction (New England Biolabs).

To generate SHFV-eGFP viruses with mutations in the ORFs encoding minor structural proteins (designated “pCMV-SHFV-eGFPΔORF 2a’/2b’/3′/4′/2a/2b/3/4”), we designed primers to introduce stop codons into the respective ORFs without altering the amino-acid sequence encoded in overlapping ORFs (Table 2).

To generate pCMV-SHFV-eGFPΔORF 2b’, a codon-randomized ORF 2b’ containing eight stop codons throughout ORF 2b’ was commercially synthesized by ATUM (Newark, CA, USA). The synthesized fragment was used to replace the original ORF 2b’ by Gibson assembly.

To replace SHFV ORF 2a, 2b, 3, and 4 with ORF 2a, 2b, 3, and 4 of EAV, pCMV-SHFV-eGFP was digested with *Spe*I and *Psi*I to release the fragment containing ORF 2a, 2b, 3, and 4 of SHFV. Three fragments were amplified. Fragment 1, which contained SHFV genome nucleotides 11729-13388, was PCR-amplified with primer 5′-*Spe*I-TRS5 (5′-GGA CGA GCT GTA CAA GTA AAC TAG TCA TCA-3′) and 3′SHFV-ORF4′-EAV (5′-CAC TAA GCC CAT TAA GAA TAG AAT GCT CTA AAG ATC-3′), with pCMV-SHFV-eGFP as a template. Fragment 2, which contained the ORFs of 2a, 2b, 3, and 4 of EAV, was RT-PCR-amplified by using the Super Script III One-Step RT-PCR Platinum Taq Hifi Kit (Thermo Fisher) with primers 5′EAV-ORF2ab34 (5′-CTA TTC TTA ATG GGC TTA GTG TGG TCA CTG ATT TCA-3′) and 3′EAV-ORF 4 (5′-AGT ACA TAA TCA TAG ATA ACA TTG TTG AGC CCA ACG-3′), with EAV RNA as a template. Fragment 3, which contained SHFV genome nucleotide s14835-15328, was PCR-amplified with primers 5′SHFV-ORF 5 (5′-TGT TAT CTA TGA TTA TGT ACT TAT GTT TAG GGA GAT-3′) and 3′ SHFV-ORF5-*Psi*I (5′-GCA GTT GTA GCA AAC TTA TAA CTC AAT AAA TGG GTT-3′), with pCMV-SHFV-eGFP as a template. These three amplified fragments, together with *Spe*I- and *Psi*I-digested pCMV-SHFV-eGFP, were Gibson-assembled to generate plasmid “pCMV-SHFV-eGFP-EAV-ORF2ab34.”

### 2.4. Rescue of Recombinant SHFVs

MA-104 cells (3 × 10^5^ cells per well, 6-well plate) were transfected with 3 µg of pCMV-SHFV, pCMV-SHFV-eGFP, pCMV-SHFV-eGFPΔORF 2a/2b/3,/4/2a’/2b’/3′/4′, or pCMV-SHFV-eGFP-EAV-ORF2ab34 with 4.5 µL of lipofectamine 3000 (Thermo Fisher Scientific). At 6 h post-transfection (p.t.), transfection mixtures were removed and 3 mL of EMEM with 2% FBS was added to cells. At 48 h p.t., tissue culture supernatants (TCSs) were harvested and subjected to plaque assay for viral titer measurement as previously described [28]. For each transfected cell line, eGFP expression plasmid gWIZ-GFP (Aldevron, Fargo, North Dakota, USA) was used as a control for transfection efficiency.

### 2.5. SHFV Growth Kinetics Comparison

MA-104 cells, seeded in collagen-coated 24-well plates (1 × 10^5^ cells per well), were exposed to wild-type SHFV (ATCC), rSHFV, or rSHFV-eGFP at a multiplicity of infection (MOI) of 0.1 or 1. After 1 h of incubation at 37 °C, viral inocula were removed, and cells were washed twice with EMEM without FBS and then supplemented with EMEM with 2% FBS. At various times post-exposure (p.e.), TCSs were harvested. Viral titers were determined by plaque assay as previously described [28].

### 2.6. rSHFV-eGFP Genetic Stability Determination

To determine the stability of the inserted eGFP ORF, rSHFV-eGFP was passaged five times in MA-104 cells. Briefly, MA-104 cells were exposed to rSHFV-eGFP (defined as Passage 0) at an MOI of 1. At 48 h p.e., TCSs were collected (Passage 1), and virus titers were determined by plaque assay as previously described [28]. Fresh MA-104 cells were then exposed to Passage 1 TCSs at an MOI of 1 to generate Passage 2. This process was serially repeated until Passage 5.

### 2.7. SHFV Titration

SHFV titers were quantified by plaque assay as previously described [28]. Briefly, confluent monolayers of MA-104 cells in 6-well plates were exposed to serial dilutions of SHFV. After 1 h of incubation at 37 °C while gently rocking every 15 min, 2 mL of a 1:1 overlay consisting of 2X Gibco modified Eagle’s medium (MEM; Thermo Fisher Scientific) and 1% Seakem ME Agarose (Lonza, Rockland, ME, USA) were added to each well. At 2 days p.e., agarose overlays were removed and the cells were stained with 0.2% crystal violet staining solution. Plaques were counted manually.

### 2.8. CD163 Antibody Inhibition Assay

MA-104 cells were incubated with different concentrations (0, 2.5, 5, 10, 20, or 40 µg/mL) of goat anti-human CD163 antibody (R&D Systems, Minneapolis, MN, USA) or control goat immunoglobulin G (IgG) at 37 °C for 1 h. Treated cells were exposed to rSHFV-eGFP at an MOI of 5 at 37 °C for 1 h in the presence of antibodies. The virus-antibody inocula were then removed, the cells were washed, and EMEM plus 2% FBS was added. The cells were fixed with 10% neutral-buffered formalin (NBF; Thermo Fisher Scientific). Nuclei were stained with Hoechst 33342 (Thermo Fisher Scientific). The percentage of eGFP-positive cells was quantified with the Operetta High-Content Imaging System (PerkinElmer, Shelton, CT, USA) and analyzed with Harmony 3.1 analysis software (PerkinElmer) as previously described [28].

## 3. Results

### 3.1. Rescue and Growth Characterization of Recombinant SHFVs

**Figure 1:**
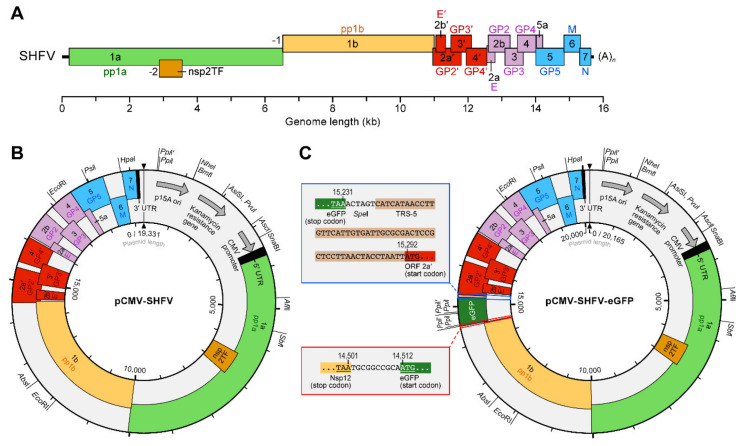
Schematics of simian hemorrhagic fever virus (SHFV) cDNA-launch infectious clones. (**A**) The SHFV genome is a linear, unsegmented, polyadenylated, positive-sense RNA. Open reading frames (ORFs) are shown as colored rectangles. ORF1a is translated directly from the genomic RNA into polyprotein 1a (pp1a). A -2 programmed ribosomal frameshift results in nonstructural protein 2 transframe (nsp2TF), whereas a -1 frameshift fuses ORF1a and ORF1b, resulting in polyprotein 1ab (pp1b). pp1a and pp1ab are co- and post-translationally cleaved to yield numerous SHFV nonstructural proteins (nsps). The remaining ORFs encode the SHFV structural proteins, which are translated from a nested set of subgenomic RNAs. E, envelope protein; GP, glycoprotein; M, matrix protein; N, nucleocapsid protein. (**B**) Constructed plasmid pCMV-SHFV, encoding wild-type SHFV. (**C**) Constructed plasmid pCMV-SHFV-eGFP, encoding SHFV-eGFP.

To construct a DNA-launch infectious clone of SHFV, we cloned the cDNA of the entire genome of SHFV variant NIH LVR42-0/M6941 (GenBank #AF180391; RefSeq #NC_003092), flanked by a CMV immediate early enhancer-containing promoter at the 5′ end and the hepatitis delta virus 1 (HDV-1) ribozyme sequence at the 3′ end to ensure generation of authentic 3′ genome sequences, into plasmid pACYC177 (“pCMV-SHFV-NC_003092.2”). However, transfection of this plasmid into MA-104 cells never resulted in successful SHFV rescue. Suspecting that the GenBank sequence may be erroneous, we sequenced the genome of a commercially available SHFV variant NIH LVR42-0/M6941 (GenBank #KM373784.1) [16] and discovered several discrepancies (Table 1). We introduced most of these discrepancies into pCMV-SHFV-NC_003092.2, yielding pCMV-SHFV, and created a derived plasmid thereof, pCMV-SHFV-eGFP, encoding eGFP between ORF 1b and ORF 2a’ (Figure 1).

**Figure 2:**
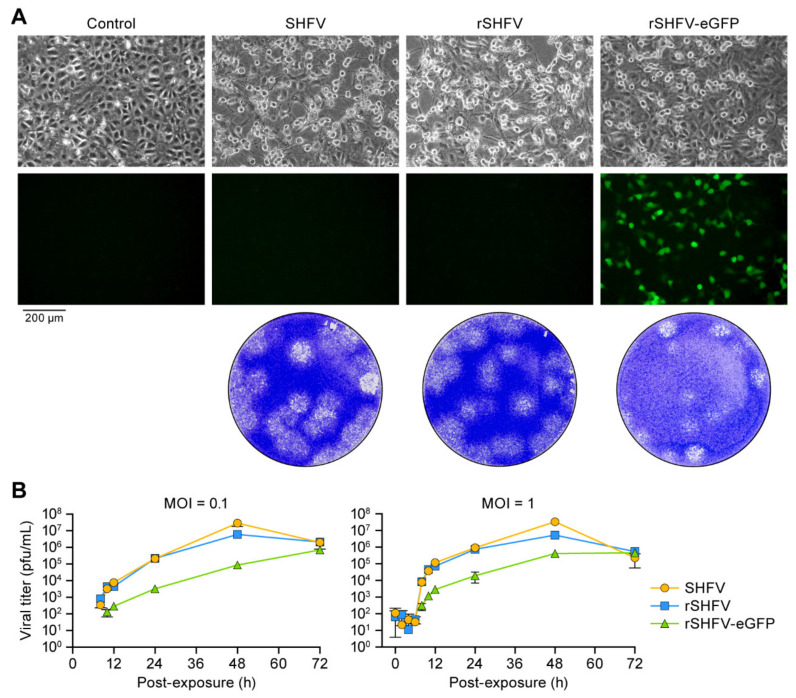
Growth kinetics of wild-type simian hemorrhagic fever virus variant NIH LVR42-0/M6941 (SHFV), recombinant wild-type SHFV (rSHFV), and rSHFV expressing enhanced green fluorescent protein (rSHFV-eGFP). (**A**) MA-104 cells were mock-exposed (control), exposed to SHFV (multiplicity of infection (MOI) of 0.1), transfected with pCMV-SHFV, or transfected with pCMV-SHFV-eGFP. Top row: Microscopic images reveal the cytopathic effect caused by SHFV, rSHFV, and rSHFV-eGFP infection at 16 h post-exposure/transfection. Middle row: Expression of eGFP was observed using epifluorescent microscopy. Bottom row: plaque morphologies of the three viruses. (**B**) MA-104 cells were exposed to all three viruses at MOIs of 0.1 (left) and 1 (right), tissue culture supernatants were harvested at the indicated time points post-exposure, and virus titers were determined by plaque assay. Graphs of viral titers represent the means ± the standard deviations of triplicate samples from one of two independent experiments.

To rescue both wild-type (rSHFV) and eGFP-expressing SHFV (rSHFV-eGFP), we transfected MA-104 cells directly with pCMV-SHFV or pCMV-SHFV-eGFP. At 16 h p.t., we observed cell rounding and detachment in plates transfected with either plasmid reminiscent of the cytopathic effect (CPE) characteristic of SHFV infection [28] (Figure 2A). Under fluorescence microscopy, green fluorescent signal was observed in pCMV-SHFV-eGFP-transfected but not pCMV-SHFV-transfected or SHFV-exposed cells, indicating the expression of eGFP from the transfected plasmid (Figure 2A). Cell culture supernatants were harvested from these transfected cells at 48 h p.t., genomic sequences of rSHFV and rSHFV-eGFP in the harvested supernatants were validated by high-throughput sequencing, and standard plaque assays were performed after transferring the supernatants onto fresh MA-104 cells to demonstrate virus growth (Figure 2B). These results indicate that viable viruses (rSHFV and rSHFV-eGFP) were recovered from the plasmids. As expected, the growth kinetics of SHFV and rSHFV were almost identical at both tested MOIs (0.1 and 1), reaching peak titers of 1 × 10^7^ PFU/mL. The titer of rSHFV-eGFP virus was approximately 10-fold lower than that of SHFV or rSHFV, suggesting that the inserted eGFP-encoding cassette attenuated the virus (Figure 2B).

### 3.2. Identification of New SHFV-Permissive Cell Lines

**Figure 3:**
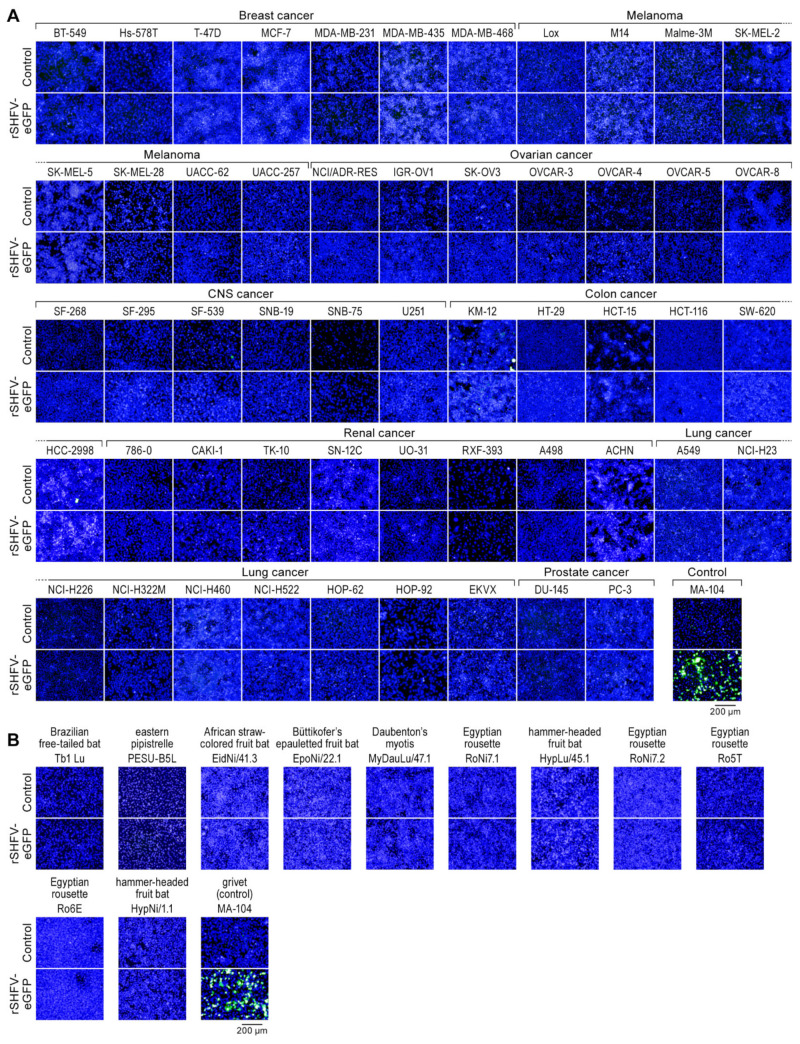
Cellular tropism of simian hemorrhagic fever virus (SHFV): Recombinant wild-type SHFV expressing enhanced green fluorescent protein-expressing (rSHFV-eGFP) does not infect human or bat cells. (**A**) 53 adherent cells of the human NCI-60 cancer cell line panel and (**B**) 11 bat cell lines were exposed to rSHFV-eGFP at a multiplicity of infection of 3. Shown are high-content images of mock-exposed controls and virus-exposed cells counterstained with Hoechst33342 at 72 h or 24 h post-exposure for positive control MA-104 cells.

We performed several proof-of-principle experiments to demonstrate the usefulness rSHFV-eGFP (Passage 1) as a tool for the identification of novel SHFV-permissive cell lines. We exposed 53 adherent cell lines of the human NCI-60 cancer cell line panel and 11 bat cell lines to rSHFV-eGFP, using MA-104 cells as a control. None of these cell lines replicated rSHFV-eGFP (Figure 3).

**Figure 4:**
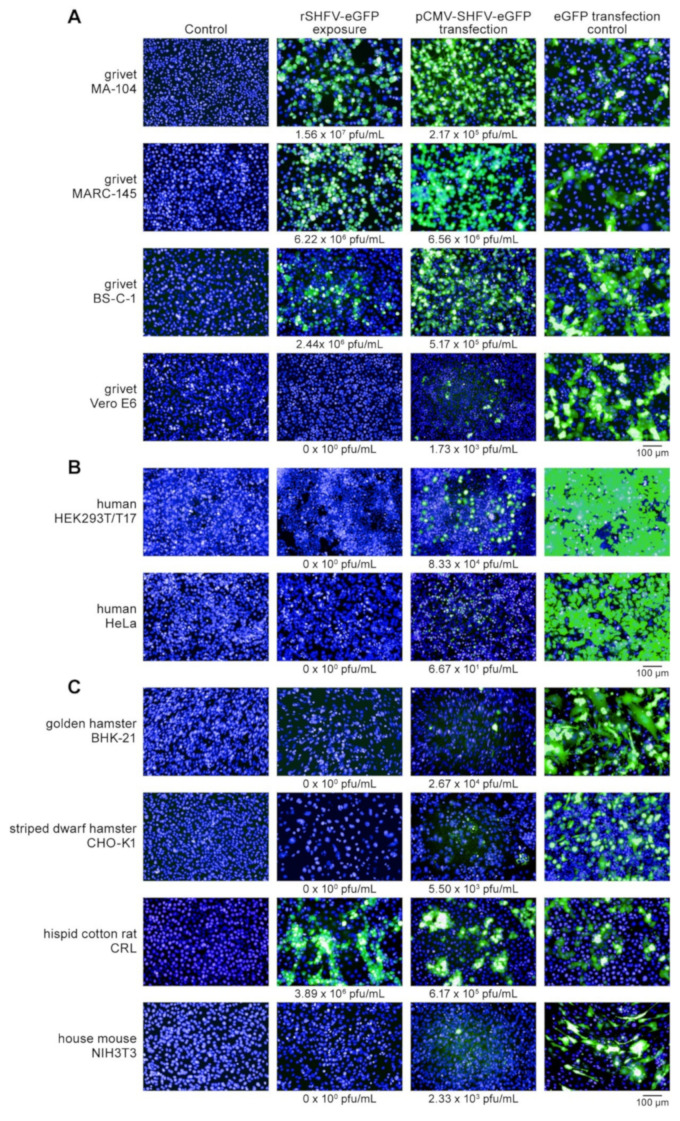
Cellular tropism of simian hemorrhagic fever virus (SHFV): Recombinant wild-type SHFV expressing enhanced green fluorescent protein-expressing (rSHFV-eGFP) infects hispid cotton rat lung (CRL) cells. (**A**) Grivet cells, (**B**) human cells that are not part of the human NCI-60 cancer cell line panel, and (**C**) rodent cells were exposed to rSHFV-eGFP at a multiplicity of infection of 3. Tissue culture supernatants were harvested at 72 h post-exposure, cells were counterstained with Hoechst 33342, and high-content images of exposed cells or mock-infected cells were taken. Supernatants of virus-exposed cells were also subjected to plaque assay; obtained titers are printed beneath the images. Uninfected cells were also transfected with pCMV-SHFV-eGFP or a control plasmid expressing eGFP only. At 48 h post-transfection, cells were counterstained with Hoechst 33342, high-content images were taken, supernatants were harvested, and viral titers were measured by plaque assay; obtained titers are indicated beneath the images.

**Figure 5:**
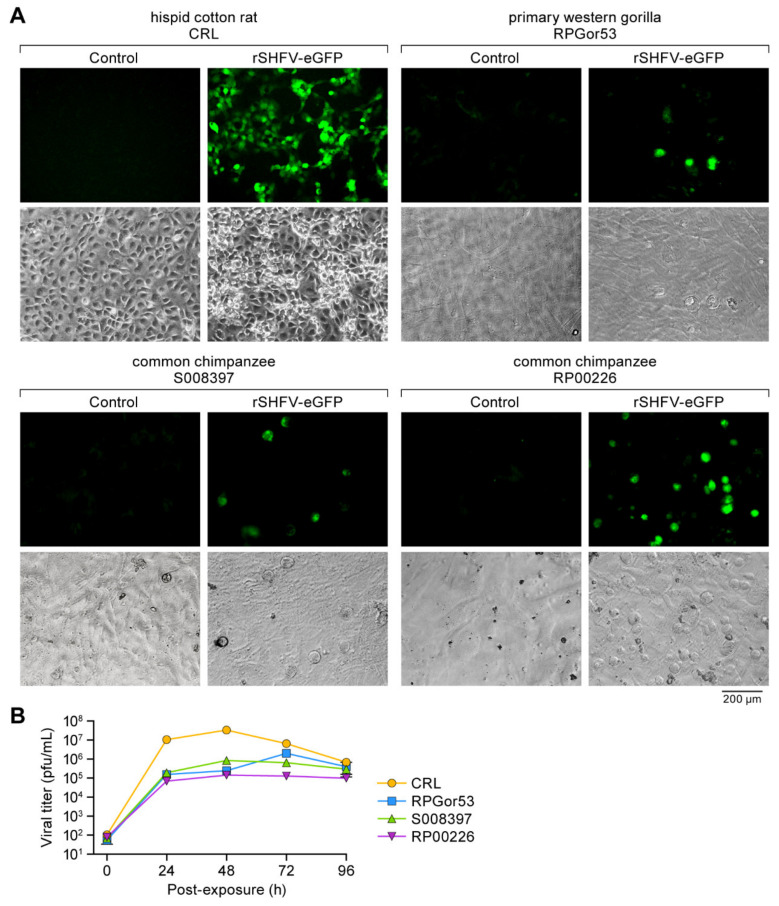
Cellular tropism of simian hemorrhagic fever virus (SHFV): Recombinant wild-type SHFV expressing enhanced green fluorescent protein-expressing (rSHFV-eGFP) infects hispid cotton rat lung (CRL) cells, western gorilla RPGor53 primary fibroblasts, and chimpanzee S008397 and RP00226 primary fibroblasts. (**A**) CRL cells, western gorilla primary fibroblasts, and common chimpanzee primary fibroblasts were exposed to rSHFV-eGFP at a multiplicity of infection (MOI) of 3. eGFP expression and cytopathic effect were observed at 24 h post-exposure. (**B**) Cells were exposed to wild-type SHFV at an MOI of 3. Tissue culture supernatants were harvested at the indicated time points post-exposure, and virus titers were determined by plaque assay. Graphs of viral titers represent the means ± the standard deviations of triplicate samples from one of two independent experiments.

We then repeated this experiment with various nonhuman primate cell lines (including MA-104, MARC-145, and BS-C-1 cells as positive controls), additional human cell lines, rodent cell lines, and primary ape fibroblasts (Figure 4 and Figure 5). As expected, all control cell lines were permissive to rSHFV-eGFP. However, most other tested cell lines did not replicate rSHFV-eGFP. Surprisingly, common chimpanzee RP00226 and S008397 primary fibroblasts, western gorilla RPGor53 primary fibroblasts, and hispid cotton rat lung CRL cells were found to be permissive to the virus (Figure 4C and Figure 5A). To further confirm the permissiveness of these cell lines to rSHFV-eGFP, we exposed them to wild-type SHFV at an MOI of 3 and measured virus growth kinetics over time (Figure 5B). The results indicated that the CRL cells are the most permissive of the four cell lines, replicating rSHFV-eGFP to 10^7^–10^8^ PFU/mL at 48 h p.e.

### 3.3. Restriction of SHFV Likely Occurs at Multiple Steps in the Cellular Host

To examine whether resistance to SHFV infection is solely determined by the inability of SHFV to enter cells, we transfected pCMV-SHFV-eGFP directly into apparently resistant grivet (Vero E6), human (293T and HeLa), and rodent (CHO-K1, BHK-21, and NIH3T3) cells as well as permissive grivet (MA-104, MARC-145, and BS-C-1) and hispid cotton rat lung CRL cells (Figure 4). As expected, we observed extensive eGFP expression in permissive cells. Only a few eGFP-expressing cells were observed in apparently resistant cell lines. Interestingly, plaque-assay data indicate that substantial viral replication occurred in 293T cells (~10^5^ PFU/mL) and limited replication occurred in other apparently resistant cell lines of distinct species. These data indicate that cells permit or restrict against SHFV infection at virion entry (susceptibility), immediately after virion entry (permissibility), or both. For instance, grivet Vero E6 cells appear to restrict SHFV infection at the virion entry step because Vero E6 cells transfected with pCMV-SHFV-eGFP plasmid resulted in limited rSHFV-eGFP replication, whereas other grivet (MA-104, MARC-145, and BS-C-1) cells take up and replicate rSHFV-eGFP directly.

### 3.4. The eGFP Expression Cassette in rSHFV-eGFP Is Unstable

**Figure 6:**
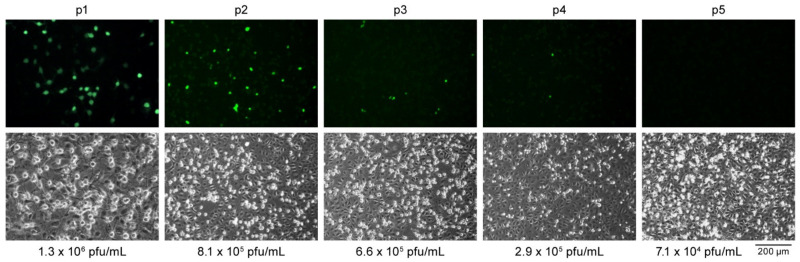
Genetic stability of recombinant wild-type simian hemorrhagic fever virus (SHFV) expressing enhanced green fluorescent protein-expressing (rSHFV-eGFP) in MA-104 cells. rSHFV-eGFP was serially passaged on MA-104 cells. Cells were exposed to rSHFV-eGFP at a multiplicity of infection (MOI) of 1, tissue culture supernatants were collected (Passage 1 [p1]) at 48 h post-exposure, and virus titers were determined by plaque assay. Fresh MA-104 cells were infected with tissue culture supernatants at an MOI of 1. This process was serially repeated through Passage 5 (p5). eGFP expression (green) was measured by epifluorescent microscopy (top row). Cells were imaged by compound microscopy (bottom row).

We investigated the stability of the eGFP reporter cassette in rSHFV-eGFP by serial passaging rSHFV-eGFP in MA-104 cells. Cells were exposed to rSHFV-eGFP at an MOI of 1. After 48 h p.e., TCSs were collected (Passage 1) and virus titers were measured by plaque assay. Fresh MA-104 cells were exposed to rSHFV-eGFP Passage 1 at an MOI of 1, and the process was serially repeated through Passage 5. The expression of eGFP in each passage, monitored by epifluorescent microscopy, dramatically decreased after Passage 2 (Figure 6), whereas plaque-assay data were largely stable. These data indicate that the virus either lost parts of or the entire eGFP cassette or rapidly accumulated deleterious mutations within it during replication.

### 3.5. The Minor Structural Protein E’ Is Dispensable for Infectious SHFV Particle Production

**Figure 7:**
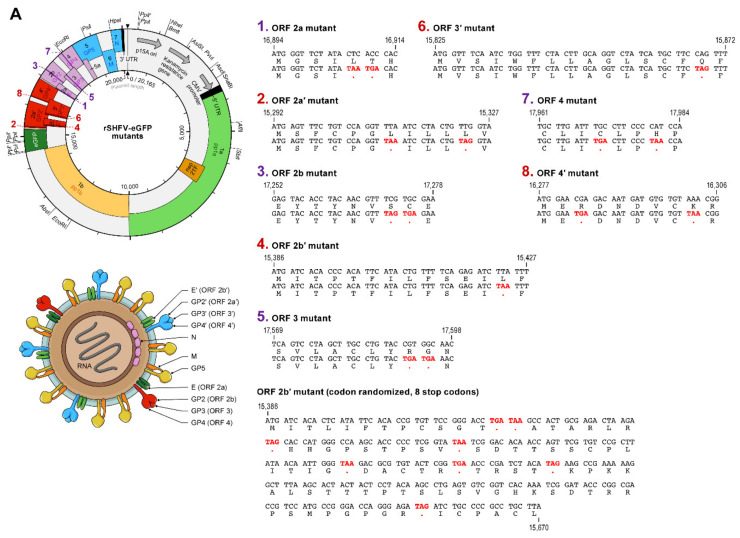

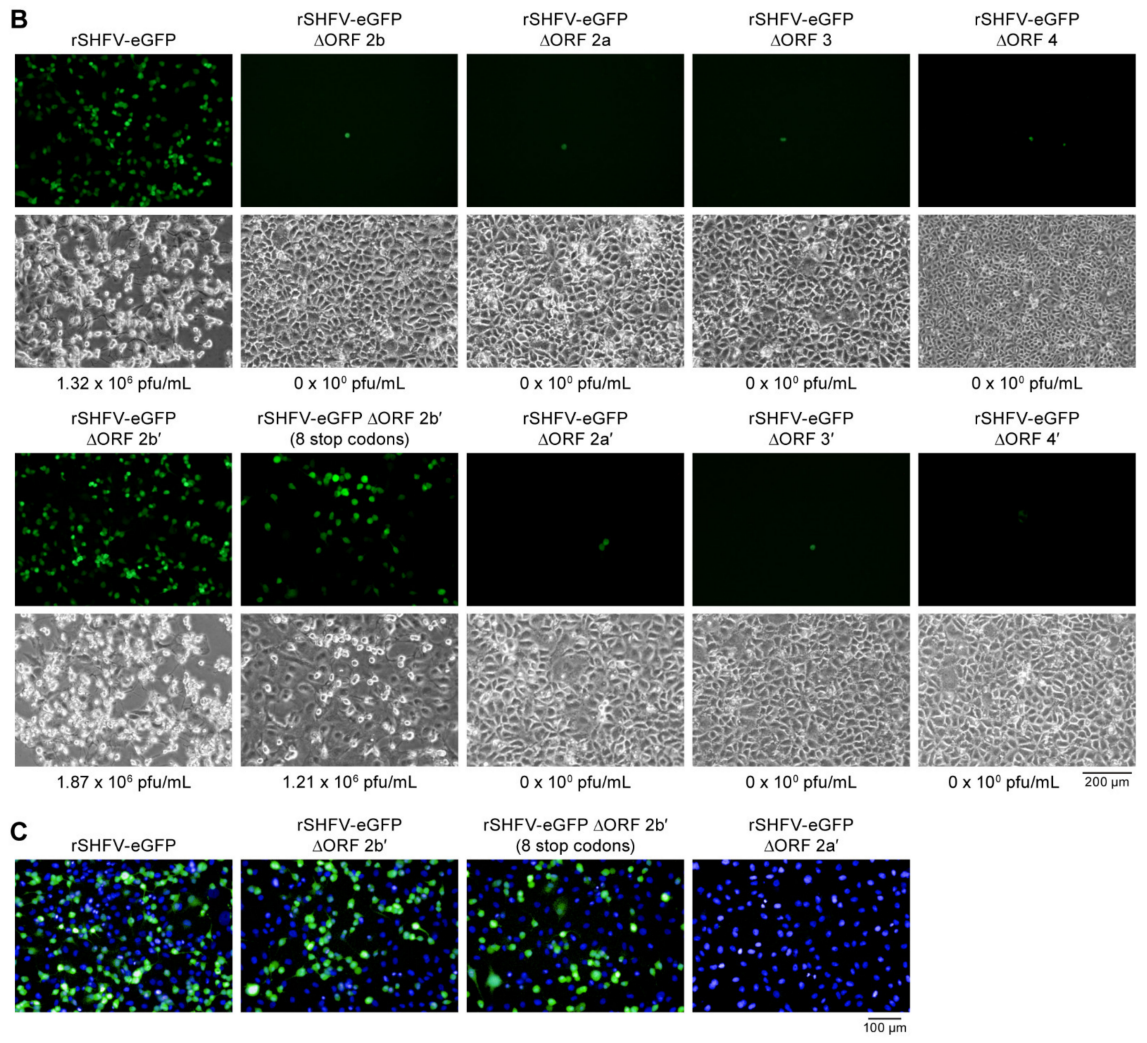
Minor structural protein E’ is dispensable for infectious simian hemorrhagic fever virus (SHFV) particle production in vitro. (**A**) To analyze the functional importance of SHFV minor structural proteins, stop codons (red) were introduced into the respective open reading frames (ORFs) of pCMV-SHFV-eGFP without altering the amino acids encoded in overlapping ORFs. (**B**) MA-104 cells were transfected with the pCMV-SHFV-eGFP mutants. Supernatants were harvested at 48 h p.t. and subjected to plaque assay. Top row: epifluorescent microscopic images of eGFP-generated fluorescence. Bottom row: Compound microscopic images of cells with plaque-assay titers printed beneath them. (**C**) MA-104 cells were exposed to supernatants harvested in (**B**) and eGFP expression was measured by epifluorescence microscopy.

Vatter et al. previously reported that each of the eight SHFV minor structural proteins is functionally important [27]. To confirm these results, we generated pCMV-SHFV-eGFP mutants that contained stop codons likely to terminate their expression without altering the amino-acid residues encoded in overlapping ORFs (Figure 7A). To evaluate whether infectious rSHFVs could be rescued from these plasmids, we transfected MA-104 cells. At 72 h p.t., TCSs were harvested and subjected to plaque assay. No CPE or eGFP expression was observed in cells transfected with plasmids preventing ORF 2b, 2a, 3, 4, 2a’, 3′, or 4′ expression and plaque assays were negative (Figure 7B), suggesting that the minor structural proteins encoded by these ORFs (E, GP2, GP3, GP4, GP2′, GP3′, and GP4′, respectively) are likely essential for SHFV propagation. However, we observed SHFV-typical CPE, eGFP expression, and titers in cells transfected with the plasmid designed to prevent ORF 2b’ expression, suggesting that the encoded protein (E’) is not required for SHFV propagation. Since this result is contradictory to results reported by Vatter et al. [27] and because it is possible that the single stop codon we introduced was somehow circumvented by the viral polymerase, we created a second pCMV-SHFV-eGFP that included eight stop codons dispersed among the entire ORF 2b’ (Figure 7A). Consistently, SHFV-typical CPE and eGFP expression were observed from cells transfected with this plasmid, and viral titers were comparable to those measured for cells transfected with prototype pCMV-SHFV-eGFP (Figure 7B). Supernatants harvested from cells transfected with pCMV-SHFV-eGFP and pCMV-SHFV-eGFP mutants containing stop codons to prevent expression of GP2′ and E’ were used to expose fresh MA-104 cells to evaluate infectious particle production. As expected, supernatants from cells transfected with the ORF 2a’ mutant plasmid did not contain infectious particles, whereas both ORF 2b’ mutants resulted in infectious progeny virions. These findings confirm that E’ does not play a vital role in in vitro SHFV replication in vitro.

### 3.6. Replacement of SHFV ORFs Encoding Minor Proteins E, GP2, GP3, and GP4 of SHFV with EAV Orthologs Does Not Expand SHFV Cell Tropism

**Figure 8:**
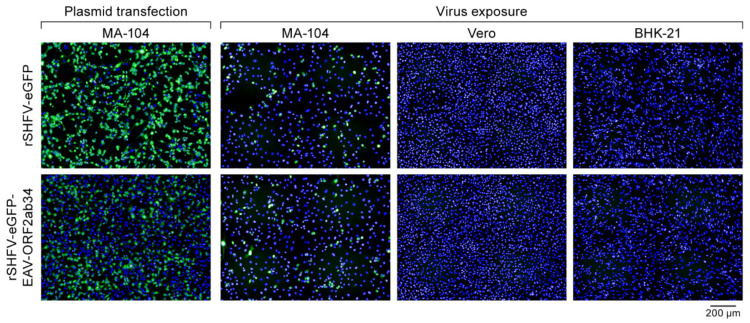
Replacement of the recombinant wild-type simian hemorrhagic fever virus (SHFV) expressing enhanced green fluorescent protein-expressing (rSHFV-eGFP) open reading frames (ORFs) encoding the minor structural proteins E, GP2, GP3, and GP4 with ORFs encoding presumed EAV orthologs does not expand SHFV cell tropism. MA-104 cells were transfected with a plasmid encoding rSHFV-eGFP control (top) or chimeric rSHFV-eGFP (bottom). At 48 h post-transfection, eGFP expression was measured by epifluorescence microscopy. Supernatants (containing rSHFV-eGFP or rSHFV-eGFP-EAV-ORF2ab34) were harvested and used to expose fresh MA-104, Vero, and BHK-21 cells at a multiplicity of infection of 1. Success of infection was measured by epifluorescent microscopy.

PRSSV-1 and PRSSV-2 have highly restricted cell tropisms, similar to SHFV [44,45,46,47]. On the other hand, EAV is promiscuous, infecting numerous cell types [48]. The EAV minor structural proteins E, GP2b, GP3, and GP4, considered to be orthologs of PRRSV-1/2 and SHFV E, GP2, GP3, and GP4, respectively, appear to be major determinants of EAV cell tropism [49]. A chimeric recombinant PRRSV-2 encoding the four EAV minor envelope proteins instead of the PRRSV-1 orthologs lost the ability to infect PRRSV-1-permissive porcine alveolar macrophages. Instead, this chimeric virus gained the ability to infect PRRSV-resistant but EAV-permissive BHK-21 and Vero cells [49]. In contrast to EAV and PRRSV-1/2, SHFV and other simarterivirins encode a second set of presumed minor structural proteins: E’, GP2′, GP3′, and GP4′. As a further proof-of-principle experiment to demonstrate the usefulness of an SHFV infectious cDNA clone, we replaced SHFV ORFs 2b, 2a, 3, and 4 in pCMV-SHFV-eGFP with the corresponding EAV ORFs, expecting that this replacement would change the tropism of the resulting rSHFV-eGFP-EAV-ORF2ab34 from that of SHFV to that of EAV. We successfully rescued the virus from MA-104 cells transfected with the chimeric plasmid (Figure 8). Interestingly, rescued rSHFV-eGFP-EAV-ORF2ab34 continued to infect MA-104 cells but not Vero or BHK-21 cells (Figure 8).

### 3.7. CD163 Is a Crucial SHFV Cell Entry Factor

**Figure 9:**
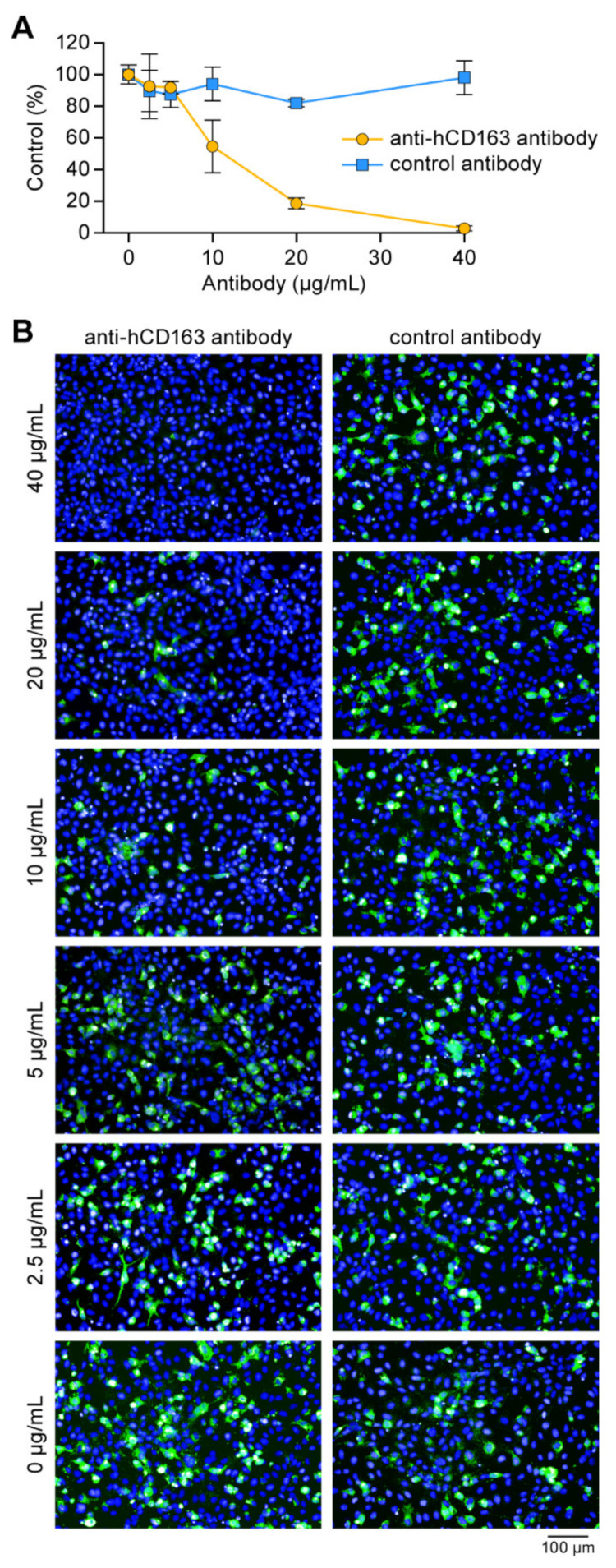
CD163 is a crucial simian hemorrhagic fever virus (SHFV) cell entry factor. (**A**) MA-104 cells were incubated with different concentrations of goat anti-human CD163 antibody or control goat immunoglobulin G (IgG) at 37 °C for 1 h. The treated cells were exposed to recombinant wild-type SHFV expressing enhanced green fluorescent protein-expressing (rSHFV-eGFP) at a multiplicity of infection of 5 at 37 °C for 1 h in the presence of antibodies. The percentage of rSHFV-eGFP-infected cells in the presence of increasing goat anti-CD163 antibody (yellow) and control goat IgG control (blue) is indicated at 48 h post-exposure. Error bars indicate the standard deviation of triplicate samples from one of two independent experiments. (**B**) Representative images of the experiment described in (**A**).

CD163 plays an important, although still relatively undefined, role in PRRSV-1/2 particle entry into host cells [28,50]. We previously demonstrated that CD163 plays a similar role in SHFV particle entry: As measured by plaque assays, SHFV-permissive cells pre-incubated with an anti-human CD163 (anti-hCD163) antibody and then exposed to wild-type SHFV produced significantly fewer progeny virions than cells treated with a mock antibody [28]. This observation suggested that CD163 may be an SHFV receptor. To confirm these results, MA-104 cells were incubated with increasing concentrations of a specific anti-hCD163 antibody and then exposed to rSHFV-eGFP. At 48h p.e., the percentage of eGFP-positive cells was measured by high-content imaging. Consistent with the previously published plaque-assay data, the percentage of rSHFV-eGFP-infected cells was dramatically reduced by anti-hCD163 antibody treatment (Figure 9).

## 4. Discussion

SHFV is a neglected pathogen. Consequently, knowledge of the molecular biology and virology of SHFV is extremely limited. The functions of most of SHFV’s identified nonstructural and structural proteins are yet to be elucidated, and a relatively recent study indicated that the proteome of SHFV is much more complex than currently appreciated [24]. One reason for the lack of understanding of this important nidovirus [15] is the absence of specific reagents for SHFV manipulation and detection. There are no commercial (or otherwise easily available) antibodies that are suitable for standard immunofluorescence assays, and probes developed for SHFV detection using RNAscope in situ immunohistochemistry [22] are relatively expensive and labor-intensive.

One way of circumventing the current shortcomings in SHFV detection would be the use of a reporter protein-encoding SHFV. The development of such a recombinant virus requires the establishment of cDNA clones from which viruses can be rescued. Previously, Vatter et al. developed such a system for SHFV variant NIH LVR42-0/M6941; the SHFV genome was cloned into a large capacity plasmid vector (pACYC184 (V)) that, after linearization, served as a template for in vitro transcription of genomic RNA, which was then transfected into MA-104 target cells [27]. We aimed at simplifying this system by circumvention of the linearization and in vitro transcription step, in part because performing this step requires specialized reagents or kits and in part because working with RNA in general presents distinct challenges. Following a previous strategy used for PRRSV-1 [42,43], we established a DNA plasmid containing the SHFV genome that can be transfected directly into target cells without prior manipulation for successful rSHFV rescue. Further following the PRSSV-1 strategy, we modified the SHFV genome in the DNA plasmid to encode eGFP in a separate expression cassette immediately upstream of the SHFV structural ORFs and demonstrated successful rescue of rSHFV-eGFP.

Next, we used this virus to demonstrate that rSHFV-eGFP is a valuable, easy-to-use tool to identify novel SHFV-permissive cell lines. Our initial results supported the notion that human cells are generally not permissive to SHFV multiplication. Given the fastidious cell tropism of SHFV, our discovery of primary common chimpanzee and gorilla fibroblast and a hispid cotton rat lung (CRL) cell lines as SHFV-permissive cells was surprising. These findings indicate that SHFV may not be as host-restricted as previously thought and infer that future experiments should include a broader panel of rodent and nonhuman primate cell lines to identify cell lines that are not only permissive to SHFV but also easy to maintain and manipulate and replicate SHFV to high(er) titers. Second, we demonstrated that some cell lines, including human, are not susceptible to rSHFV-eGFP infection after direct exposure but they support rSHFV-eGFP replication and particle egress if the infectious clone plasmid is transfected directly into them. Although thus far limited in scope, these data indicate that, for instance, human cells restrict SHFV replication in at least two points: the cell entry step (because SHFV replicates in some cells after entry restriction is circumvented) and the step post-entry (because SHFV does not replicate in some cells even after entry restriction is circumvented). Third, we used the same strategy previously published by Vatter et al. to evaluate the functional importance of the eight SHFV minor structural proteins [27]: We attempted to knock out each protein by introduction of stop codons into the respective ORFs. We confirmed the data from Vatter et al., i.e., that minor structural proteins E, 2, 3, 4, 2′, 3′, and 4′ are likely required for the SHFV propagation. Unexpectedly, though, we successfully rescued rSHFV-eGFP containing one and eight stop codons in ORF 2b’, indicating that expression of E’ is dispensable for SHFV propagation. Fourth, in an already-published study, our cDNA clone was used to characterize −2/−1 programmed ribosomal frameshifting during SHFV ORF 1a translation [51].

Together, these data build confidence in our system being a useful tool for the study of SHFV molecular biology. Of course, the system will require further optimization. The current location of the eGFP-expressing cassette is not ideal as even a few passages of rSHFV-eGFP resulted in loss of fluorescence, likely due to a compromise of the eGFP-encoding ORF. A study by Di et al. indicated that such compromise can be circumvented by shuffling the ORF to different locations in the SHFV genome, replacing the type of encoded reporter, or both [52]. In addition to the creation of a more stable reporter-encoding SHFV for advanced in vitro studies, it would be useful to determine the pathogenicity of such a virus in vivo in a macaque host. A virulent reporter virus would open the doors for sophisticated pathogenesis studies and, in the long run, simplify the evaluation of potential medical countermeasures against SHFV.

## 5. Conclusions

In conclusion, we developed a DNA-launch recombinant SHFV infectious clone system and, using proof-of-principle experiments, demonstrated the potential application of this system to study various aspects of the SHFV lifecycle.

## Figures and Tables

**Table 1:** Nucleotide differences between simian hemorrhagic fever virus (SHFV) variant NIH LVR42-0/M6941 genomes GenBank.

Nucleotide Position in Genome	SHFV Variant NIH LVR42-0/M6941 (GenBank #AF180391)	SHFV Variant NIH LVR42-0/M6941 Isolate KS_06_17_11 (GenBank #KM373784)	Amino-Acid Residue Change	Affected Protein
511	C	T	P101L	Nsp1α
849	G	T	V214F	Nsp1β
1658	G	A	R483R (silent)	Nsp1γ
2503	G	C	G765A	Nsp2
3726	C	~	10 amino-acid frameshift	Nsp2
3757	~	A		Nsp2
3785	A	G	E1192E (silent)	Nsp2
4277	T	G	L1356L (silent)	Nsp3
4895	A	G	E1562E (silent)	Nsp4
5575	G	T	G1789V	Nsp5
5695	G	A	R1829H	Nsp5
5704	A	T	K1832I	Nsp6
5707	C	G	P1833R	Nsp6
5727	A	T	I1840F	Nsp6
6088	G	C	G1960A	Nsp7
8276	T	G	S2879A	Nsp9
8340	G	T	W2900L	Nsp9
10359	T	C	L3373S	Nsp11

Nsp = nonstructural protein; Grey shading = silent mutations.

**Table 2:** Primers used to generate the minor structural protein mutants (introduced stop codons are written in red).

Open Reading Frame (ORF)	Primer Name	Primer Sequence (5′→3′)
ORF 2a	5′SHFV-2a 16906	TCT ATA TAA TGA CAC ATC ACT ACA GCC TTC CAC
	3′ SHFV-2a-16906	AGT GAT GTG TCA TTA TAT AGA ACC CAT TAA GAA
ORF 2b	5′SHFV-2b-17266	TAC AAC GTT TAG TGA GAA CCC AGT TCT TTC ACC CTC GAC
	3′SHFV-2b 17598	GTT TTG GTC AAT GAC AAC GTT
ORF 3	5′SHFV-ORF3-*Acl*l-17266	GCT GAG TAC ACC TAC AAC GTT
	3′SHFV-ORF3-*Acl*l-17598	GAC AAC GTT TCA TCA GTA CAG GCA AGC TAG GAC TGA
ORF 4	5′SHFV-ORF4-17970	TTG ATT TGA CTT CCC TAA CCA AAT CGC ACT TCT
	3′SHFV-ORF4-17970	TGG TTA GGG AAG TCA AAT CAA GCA AGT TTG ATC
ORF 2a’	5′SHFV-2a’-15321	TAA ATC CTA CTG TAG GTA CTC CTT AAA CCA GTC GAC TCT TTT GAT TTC TTT
	3′SHFV-2a’-15321	GAG TAC CTA CAG TAG GAT TTA ACC TGG ACA GAA ACT CAT AAT TAG GTA GTT AAG GAG CGG
ORF 2b’	5′SHFV-2b’-15436	GAG ATC TAA TTT CCC ATT GCG AAA CAA AAA TTG CGC CGT
	3′SHFV-2b’-15436	CGC AAT GGG AAA TTA GAT CTC TGA AAA CAG TAT GAA TGT
ORF 3′	5′SHFV-3′-15868	TCA TGC TTC TAG TTT TGC CAC GTT TAT TGC ACC
	3′SHFV-3′ 15868	GTG GCA AAA CTA GAA GCA TGA TAG ACC TGC AAG
ORF 4′	5′ SHFV-4′-16283	TGA GAC AAT GAT GTG TGT TAA CGG CAC TGT AGT
	3′SHFV-4′-16283	TTA ACA CAC ATC ATT GTC TCA TTC CAT GAT TGC
